# Effect of Polishing Systems on the Surface Roughness of Nano-Hybrid and Nano-Filling Composite Resins: A Systematic Review

**DOI:** 10.3390/dj9080095

**Published:** 2021-08-12

**Authors:** Robinson Jaramillo-Cartagena, Eider J. López-Galeano, Federico Latorre-Correa, Andrés A. Agudelo-Suárez

**Affiliations:** Faculty of Dentistry, University of Antioquia, Medellín 050010, Colombia; robinjc20@gmail.com (R.J.-C.); eider_genius@hotmail.com (E.J.L.-G.); latorre.federico29@gmail.com (F.L.-C.)

**Keywords:** composite resins, dental polishing, finishing, dental, systematic reviews as topic

## Abstract

Background: The polishing of surface roughness is an important characteristic of composite resins and is directly related to the longevity of the restoration and patient comfort. Different polishing systems utilize different protocols, as reported in the literature. This systematic review (SR) aimed to synthesize and analyze the available scientific evidence about the effect of polishing systems on the surface roughness of nano-hybrid and nano-filling composite resins. Methods: The study protocol of this SR was registered at the International Prospective Register of Systematic Reviews -PROSPERO- (CRD4201705653). A search was conducted in PubMed-Medline, Scopus, LILACS, EMBASE, for the period 2007–2020. Quality appraisal and a descriptive analysis of the papers that met the inclusion criteria were conducted. Results: 18 records were included. Seven polishing systems (PS) of one step were found, seven PS of two steps, eight PS of three steps, three PS of four steps, and four PS of five steps. Polishing protocols (PP) varied, with application times ranging from 10 s to 60 s with speeds between 10,000 and 30,000 RPM. Regarding composition, the aluminum oxide was one of the most important components used to achieve a smooth surface. Conclusions: Multistep polishing systems were the most effective (i.e., Astropol and Sof-Lex Discs).

## 1. Introduction

Composite resins were introduced to dentistry more than 50 years ago [1]. This kind of dental material has presented disadvantages, such as high pigmentation and accelerated wear [2]. However, these materials are universally accepted for direct restorations due to their aesthetics, adhesion capacity (including an adhesive system), longevity, and thermal insulation (especially when light cured composite resins are considered) [2,3,4]. The mechanical properties of composite resins are related to their long term success [2], and this depends on their microstructure [5]. Therefore, the amount of load, size, morphology, and distribution of the filler particles are essential for material selection [1,2]. The most important current changes consist in reducing the size of the filler particles, thus obtaining materials that are easier to use and more effective [1,6]. Surface hardness is influenced by the degree of surface roughness and predicts wear resistance and ability to grind the opposing tooth [7,8].

Polishability is an important characteristic of composite resins: a smooth surface gives the restoration better aesthetics and comfort to the patient, reduces discoloration, facilitates hygiene [9,10,11,12], decreases bacterial adhesion [10], and reduces tissue inflammation, secondary caries, biofilm retention [9,10,13,14] and the risk of fracture [11]. Similarly, the use of composites is relevant in other clinical fields, such as orthodontics, where polishing systems play an important role after the treatment to ensure aesthetics and to reduce associated dental or periodontal complications [15,16,17,18]. A polished surface reduces initial bacterial adhesion and the development of biofilm in the restoration and the adjacent dental surfaces [19]. The surface roughness threshold for bacterial retention is 0.2 μm; below this, a reduction in plaque accumulation could be expected [20]. Changes of even 0.3 μm in the surface finish can be easily detected by the tongue [8,12]. Finishing procedures remove excess material with particles larger than 25 μm, while polishing procedures remove particles smaller than 25 μm [12]. At present, it is believed that composite resins with nano-filled particles obtain a better polish and shine after being subjected to different polishing processes. However, in a systematic review carried out by Kaizera et al. [21], it was concluded that there are no statistically significant differences in the polishing and brightness of composite resins with nano-filled particles vs composite resins with hybrid or nano-hybrid particles.

The classification of composite resins has mostly focused on filler-size distribution, filler content or composition [1,22]. For instance, conventional materials (or macro-filled, containing particles larger than 1 μ). However, rather than “microfills” or “nanofills”, containing only micro or nanoparticles, modern resin composites are considered a “hydrid” category, commonly named “nanohybrids” to refer to materials containing a fraction of nanoparticles (<100 nm) and of sub-micron particles (≤1 μm, typically averaging 0.5–1.0 μm) [1,6,22]. Nanofill composite resins contain only nanoscale particles, which provide a more polished surface, less shrinkage, color stability, and superior aesthetics [20,23].

To obtain an optimum polishing and shine of the composite resins, whose particles vary in hardness, shape and size, it is necessary to subject the material to abrasion processes [9,11]. Currently, an attempt has been made to determine which abrasion system provides the most polished surface for the composite resins, and several methods have been introduced without reaching a consensus that demonstrates which is the best. Some polishing methods or systems are: silicone discs, tungsten carbide burs, rubber cups, abrasive belts, and polishing pastes. These are available in one step polishing systems and multistep polishing systems [8,13,19,24,25]. 

Research in the field of the polishing and shine of composite resins is justified by the need to create a restored dental surface with optimal aesthetics and biological and functional properties in the patient. The amount of in vitro research that currently exists suggests the need to work on the comparison of the results obtained which will guide the improvement in behaviors and parameters to obtain better polished restorations [21].

Accordingly, this study aimed to conduct a systematic review that seeks to analyze and synthesize the available scientific evidence on the polishing systems of nano-hybrid and nano-filling composite resins to guide the clinician in obtaining an optimal polish that favors the aesthetic expectations of the patient and improves dental and periodontal health.

## 2. Materials and Methods

### 2.1. Study Protocol and Registration 

The study was approved by the Ethical Committee of the Faculty of Dentistry at the University of Antioquia (Act 2/2017). In addition, the protocol of the systematic review was registered at the International Prospective Register of Systematic Reviews -PROSPERO- (CRD42017056536). This paper was written according to the PRISMA statement for systematic reviews and meta-analysis [26].

### 2.2. PICOs Question and Eligibility Criteria

The PICOs (population/participants, intervention, control, outcome and study design) strategy to formulate a focused question was used as follows: What is the scientific evidence related to in vitro studies (s) about the effect of different polishing systems (I-C) on the surface roughness (O) in nano-hybrid and nano-filling composite resins (P)? According to this question, we considered papers that accomplished these criteria:

- Study design/eligibility criteria: in vitro studies published in Spanish, English, and Portuguese between January 2007 and December 2020 (At beginning of this study and according to the dates of approval of this protocol in PROSPERO, the research team decided to include the searches period 2007–2017. Later, to update the information, we applied the search period by including 2018–2020. We excluded other formats, such as theoretical reviews, interventions, observational or analytic studies, critical and theoretical essays and clinical guides.
- Population/participants: polishing systems for composite resins (nano-filling/nano-hybrid composite resins).
- Intervention/Control: different types of nano-filling/nano-hybrid composite resins.
- Outcome: the degree of surface roughness of nano-filling/nano-hybrid composite resins subjected to different polishing system.

### 2.3. Search Strategy and Data Extraction

Four electronic databases in health sciences were searched: PubMed-Medline, Scopus, LILACS (Latin American scientific literature in health sciences), EMBASE (The Excerpta Medica Database). In addition, grey literature (Google Scholar) searches through the reference lists of articles included, and manual consultations in specialized journals were conducted. This process was led by two reviewers (R.J.C and E.J.L.G). In the first round, the title and abstract were evaluated to define potential articles. After this, duplicate references were excluded; the full texts of remaining articles were reviewed. This process was conducted manually by R.J.C and E.J.L.G. Any discrepancies were resolved by the consensus of all four reviewers. We conducted a pilot test for one database to ensure concordance in the data extraction process, with a simple concordance index of 85% (by using ten abstracts).

Different combinations of text words and thesaurus terms were used. For example, the search query for PubMed was the following: (((((“Composite Resins”[Mesh]) OR (resin* OR composite* OR restorative*))) AND ((((nanofill* OR nanostructure* OR nanocomposite* OR nanoparticle* OR nanoscale* OR submicron*))) OR (((“Nanoparticles”[Mesh]) OR “Nanocomposites”[Mesh]) OR “Nanostructures”[Mesh]))))) AND ((surface roughness) OR (rough* OR smooth* OR luster* OR gloss* OR polish* OR finish*)). For EMBASE, the search query was: ‘resin’/exp or ‘resin’ or ‘composite dental resin’/exp or ‘composite dental resin’ or (‘composite’/exp or composite and (‘resins’/exp or resins)) and (nanofill* or ‘nanostructure’/exp or nanostructure or ‘nanomaterial’/exp or ‘nanomaterial’ or ‘nanoparticle’/exp or ‘nanoparticle’ or nanoscale or ‘submicron particle’/exp or ‘submicron particle’ or submicron*) and (‘submicron particle’/exp or ‘submicron particle’ or ‘surface roughness’/exp or ‘surface roughness’ or rough* or smooth* or luster* or gloss* or polish* or finish*). For other databases, we adapted these key words. 

### 2.4. Critical Appraisal and Studies’ Analysis

Two of the authors (R.J.C and E.J.L.G) reviewed the quality for reporting in vitro studies. This process was checked by a third reviewer (A.A.A.S) who has expertise in epidemiology and systematic reviews. For this purpose, we applied a modified CONSORT checklist of 14 items for reporting in vitro studies of dental materials [27,28]. After a careful evaluation of papers, we selected those accomplishing at least 70% of all items for subsequent descriptive analysis. To guarantee the quality process, we conducted a pilot test with 5 articles, and we calculated a simple concordance index, with a score of 90%. 

### 2.5. Data Analysis

We carried out a descriptive analysis of the main characteristics of the included reviews: the first author and year of publication, country, journal, study objective(s), composite resin name^®^, type of composite resin, manufacturer^®^.

To group the results obtained in Ra from the different studies, it was decided to average the different samples using the formula “Mean of Mean” ($\bar{X}$ of $\bar{X}$):

(1) $$\bar{X}\;\mathrm{of}\;\bar{X}=\frac{C1P1+C2P2+C3P3}{C1+C2+C3}$$

In this formula, *CxPx* is mean in Ra, *P* is the mean value, and *Cx* is the quantity (samples). All values are represented with means and their standard deviation (*±SD*). 

## 3. Results

**Figure 1:**
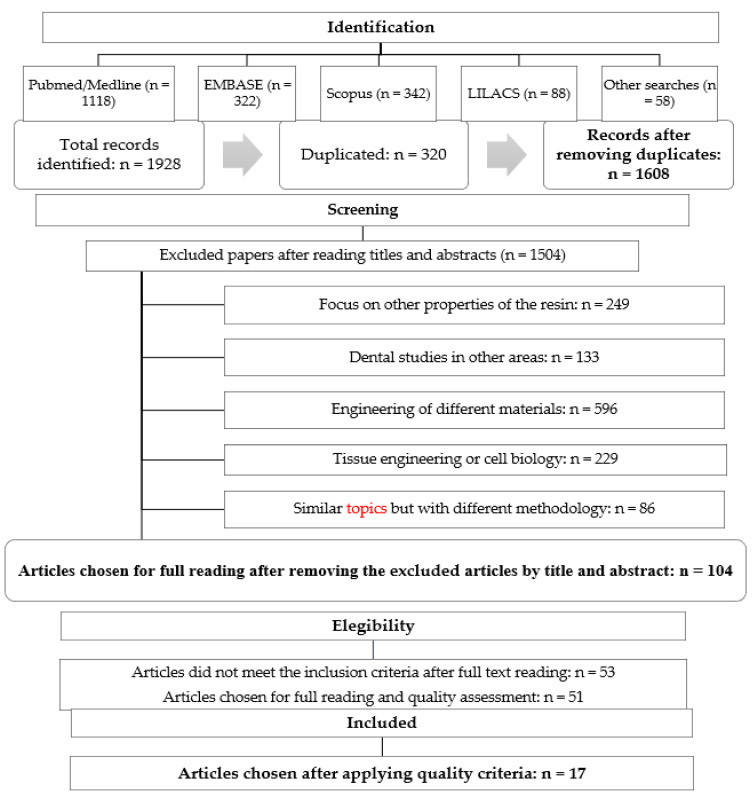
Flowchart of searching and selection process of articles for the systematic review (2007–2020).

The search in the different databases yielded a result of 1608 records (after eliminating duplicates). After the complete reading of abstracts, and application of the exclusion criteria, 51 relevant articles were found. Finally, when applying the quality criteria, 17 articles were chosen for the systematic review (Figure 1—Table 1) [3,20,29,30,31,32,33,34,35,36,37,38,39,40,41,42,43]. Following the application of the quality criteria, a minimum of 10 items from a total of 14 were defined, which represents a threshold of 71.4% [27,28].

Table 2 summarizes the information from the 17 articles selected for this review. It was found that the countries with the highest number of publications were Turkey [3,30,31,33,40,41] and Brazil [32,34,37,39,42], with six (35.3%) and five (29.4%) publications, respectively. The most commonly used composite resins were: Filtek supreme in ten studies (58.8%) [3,29,30,31,35,36,37,38,39,40], Ceram X in seven studies (41.2%) [3,20,29,30,31,33,43], and Grandio in five studies (29.4%) [3,30,31,35,40]. The most commonly used polishing systems were: Sof-Lex Discs in 14 studies (82.4%) [3,20,29,31,32,35,36,37,38,39,40,41,42,43], Enhance in combination with PoGo in 5 studies (29.4%) [32,33,34,36,37], and the PoGo system, also in 5 studies (29.4%) [3,30,31,38,43].

**Figure 2:**
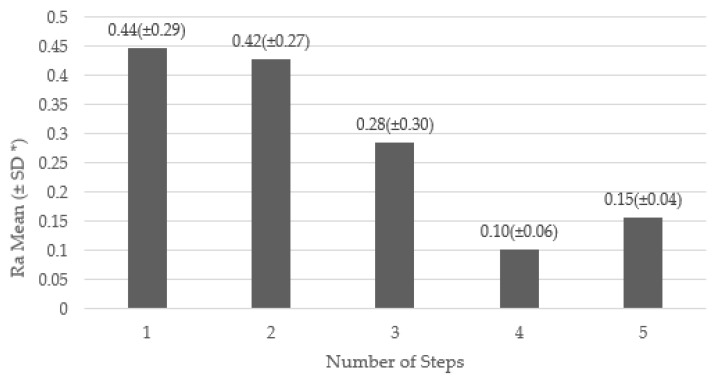
Surface roughness according to number of steps for the polishing systems (PS). Note: Each bar, listed from 1 to 5, represents the mean value (±SD) reached in Ra µm according to the number of steps of the PS. * SD = Standard Deviation.

Regarding polishing protocols (PP) and the number of steps per system, the following results were found, according to the studies included in the systematic review: seven one step polishing systems (PS) [3,30,31,32,33,35,37,38,39,40,41,43], six two step PS [32,33,34,36,37,41], eight three step PS [3,20,29,31,32,33,35,36,39,40,41,43], three four step PS [29,35,37,38,39,42], and four five step PS [42]. PP varied, with application times ranging from 10 s to 60 s with speeds between 10,000 and 30,000 rpm. This information is detailed in Table 3. When comparing the number of steps of the polishing systems with the average of the Ra of the different samples, it was found that as the number of steps increased, the Ra decreased (Figure 2).

**Figure 3:**
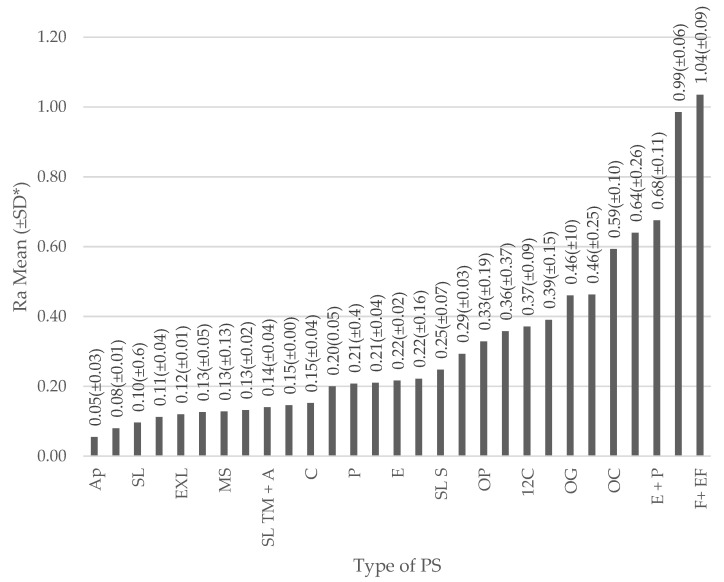
Surface roughness according to the polishing system (PS). Note: The figure represents, on the X axis, the PS and, on the Y axis, the surface roughness reached in Ra µm. The height of each bar represents the mean Ra (±SD) obtained for each PS. * SD = Standard Deviation. List of abbreviations for type of PS: Ap (Astropol), SSR (Super-snap Rainbow Technique Kit [Shofu]), SL (Sof-Lex Discs [4 SPS]), SL TM (Sof-LexTM Pop-On + felt disc associated to diamond paste [Diamond GlossTM]), EXL (EXL-695), FD E (FlexiDisc and Enamelize), MS (Mylar strip), P + F (Práxis TDV + felt disc associated to diamond paste [Diamond GlossTM]), SL TM + A (Sof-LexTM Pop-On + Astrobrush), E + CUP (Enhance + CUP shape Prisma Gloss paste), C (CompoSystem), CTD (Clearfil Twist Dia), P (Praxis TDV), P + A (Práxis TDV + Astrobrush), E(Enhance), POGO (PoGo), SL S (Sof-Lex Spiral Finishing & Polishing Wheels), F + F + D (Fine grit diamond burs 30μm and extra-fine diamond burs 20μm + Felt disks + diamond paste), OP (OptraPol), SL 3 (Sof-Lex Discs [3 SPS]), 12C (12 then 30 fluted carbide finishing bur), SR (Silicone rubber Kerr), OG (One Gloss), E + P (Enhance + PoGo), OC (Occlubrush), F + D (Felt disks + diamond paste), E + P (Enhance + PoGo + nanotechnology liquid polish [lasting touch]), L (Lasting Touch), F + EF (Fine then extrafine diamond finishing bur).

According to the PS used versus the Ra obtained on the mean for various samples (Figure 3), it was found that the polishing systems with lower Ra were, Astropol, Super-snap Rainbow Technique Kit and Sof-Lex Discs (4 SPS), with an Ra of 0.0549, 0.0799, 0.0961, respectively [20,29,35,36,37,38,39,42], and the systems with the highest Ra were Enhance + PoGo + Nanotechnology Liquid Polish (lasting touch), Lasting Touch and Fine then extra-fine diamond finishing bur, with an Ra of 0.6750, 0.9850, and 1.0350, respectively [33,40]. 

**Figure 4:**
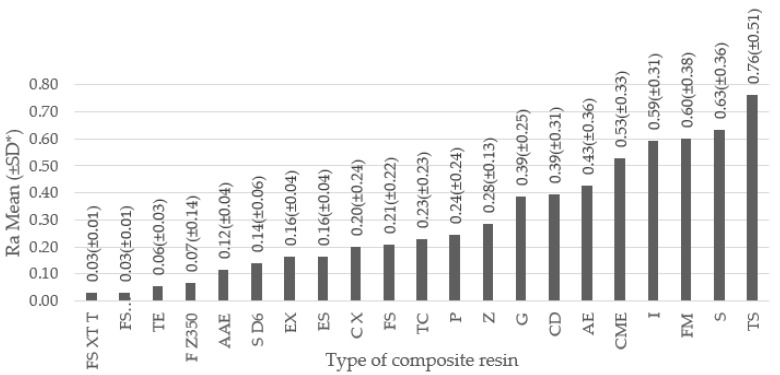
Surface roughness according to the composite resin used. The figure represents, on the X axis, the resin used and, on the Y axis, the surface roughness in Ra µm. The height of each bar represents the mean of Ra (±SD) obtained according to each composite resin. * SD = Standard Deviation. List of abbreviations for type of composite resin: FS XT T (Filtek Supreme XT [transparent shade]), FS XT D (Filtek Supreme XT [dentin shade]), TE (Tetric EvoCeram), F Z350 (Filtek Z350), AAE (Aelite Aesthetic Enamel), S D6 (Synegy D6), EX (Evolu-X), ES (Estelite Sigma), C X (Ceram X), FS (Filtek Supreme), TC (Tetric Ceram), P (Premise), Z (Zenit), G (Grandio), CD (Charisma Diamond), AE (Aelite Enamel), CME (Clearfil Majesty Esthetic), I (Ice), FM (Fill Magic^®^), S (Smile), TS (TPH Spectrum^®^).

The comparison between the composite resin type and the surface roughness (Figure 4), showed a lower Ra for Filtek Supreme XT (transparent shade), Filtek Supreme XT (dentin shade) and Tetric EvoCeram systems, with a Ra in microns of 0.0290, 0.0307, 0.0550 respectively [29], and the composite resins that showed greater surface roughness were Fill Magic^®^, Smile, TPH Spectrum^®^ with a Ra in microns of 0.5992, 0.6340, 0.7618, respectively [32,40].

## 4. Discussion

The main findings of this systematic review showed the available scientific evidence about the effect of polishing systems on the surface roughness of nano-hybrid and nano-filling composite resins through the analysis of in vitro studies. The decision to include studies expressing surface roughness results in Ra units was mainly because it was the most widely used unit of measure in most of the studies that evaluated the surface roughness of the composite resins. Other units of measure for surface roughness, such as Rz, Ry and Sa, were absent in the vast majority of studies, which was why it was decided not to use these units.

The vast majority of studies reported a control group, with the application of a pressure Mylar band on the composite resin being the most popular, with a total of 328 samples and an average of Ra 0.1281. However, Alawjali and Lui in 2013 published an in vitro study in which they demonstrate that Mylar groups generate greater color change after being subjected to different substances with pigments, and mention that the increase in discoloration can be explained by the presence of a layer rich in the composite resin matrix that is formed on the surface of the restoration [44]. It has also been shown that the polishing achieved with the Mylar strip results in surfaces with less hardness (versus abrasive polishing systems) and, consequently, greater discoloration [44]. Therefore, the removal of the composite resin matrix by finishing and polishing will allow the harder filler particles to remain in contact with the surface during polishing, producing a harder and pigment resistant surface [20].

Concerning the PP, the great heterogeneity in this regard is evidenced from the total of 11 different protocols. Besides, once the results of the studies selected were averaged, only 11 polishing systems exceeded the 0.2 µm threshold, which is considered important to reduce plaque accumulation, the possibility of secondary caries and periodontal problems [29,35,36,38,41]. The best result was obtained by the Astropol system, with an average Ra of 0.0549µm after being applied in 85 samples in the studies of Antonson et al. in 2011 and Senawongse et al. in 2007 [29,36]. Two protocols were reported, one of 20 s and another of 60 s of application, and it was appreciated that with both protocols Astropol could be below the threshold of Ra 0.2 µm [29,36]. The Super-Snap Rainbow Technique Kit system obtained the second best performance with an Ra mean of 0.0799. However, it is important to be clear that this system was only reported by Yadav et al. in 2016 [20], and this system was applied in 10 samples. The Sof-Lex Discs four step system obtained an average Ra of 0.0961 µm after being applied in 154 samples in five of the selected articles, with the use of this system six different protocols were found, all managing to exceed the limit of 0.2 µm [29,35,37,38,42]. 

On the other hand, the three step Sof-Lex Discs system was applied, obtaining an average of 0.3577 µm in a total of 221 samples in eight studies [3,20,31,32,36,40,41,43], of the five protocols reported with this system, it is well known that to gain optimal results, each disc must be applied for a time longer than 45 s, since whenever the application time is shorter, the result will be greater than 0.2 µm. This information is important, since according to Yadav et al. [20], the prolonged application of the Sof-Lex Discs system may have a disadvantage, that the friction heat generated by the discs causes microcracks in the polymer matrix that creates a rougher surface for hybrid composites.

It should be clarified that in this systematic review, some studies used the Sof-Lex Discs as a finishing and polishing system, fully (four steps) or partially (three steps, which means, ignoring the first disk in the system stream). Accordingly, the results were grouped separately according to whether four or three steps were used in the Sof-Lex Discs PS. 

According to the results, in most systems that exceeded the 0.2 µm Ra threshold, aluminum oxide was found to be a common denominator within components which produce much smoother surfaces and this is explained by the fact that aluminum oxide has a higher hardness than most filler particles in composite resins. Otherwise, the polishing agent would only remove the soft composite resin matrix leaving filler particles protruding from the surface [29,36,40]. According to Weinstein [45], when systematically decreasing the particle size of the abrasive, a smoother surface can be achieved. Likewise, the polishing material must be smaller than the particle size of the restorative material being polished to produce better results [45]. This reveals the success of the Astropol system in most of the studies, since in its last step only abrasive particles of 0.3 µm were observed.

The number of steps of the systems that exceeded the 0.2 µm Ra threshold was between two and five. It was observed that the EXL-695 experimental disc system, with an Ra of 0.12 µm [36], and Clearfil Twist Dia, with an Ra of 0.2 µm [41], were two step systems. In the study by Antonson et al. [36], the EXL-695 system was applied only to five composite resin types with a small sample being meaningful, and in the study by Aytac et al. [41], the Clearfil Twist Dia system was applied to 40 samples with a protocol of 45 s per step. However, unlike most PS, this is not composed of aluminum oxide, but rather rubber with diamond grains, and in this case, it was shown that they exceeded the control group of the Mylar band and the three step Sof-Lex Discs, Occlubrush, Sof-Lex Spiral Finishing and Polishing Wheels systems, and obtained an Ra higher than 0.2 µm. This suggests that smooth surfaces can also be achieved with diamond abrasive particles or diamond polishing pastes using finishing and polishing systems containing these particles [41].

Considering the reduced steps, application time and elimination of the risks of cross infection, single step polishing systems have become an interesting option [36]. In this systematic review, no single step polishing system managed below the 0.2 µm Ra threshold. However, the Enhance systems with a mean Ra of 0.2167 µm after being applied in 30 samples with a 20 s PP, as is shown in the study of Nunes et al. [39], and PoGo with an Ra of 0.2218 µm after being applied in 121 samples with a PP of 10 to 30 s [3,30,31,38,43], achieved results that were close to the threshold, which means greater simplicity in the clinical protocol. When the PP was reduced to 10 s with the PoGo system, as is shown in the study by Buchgraber et al. [38], a result of an Ra of 0.560 µm was obtained. Although the one step PS mostly offer results somewhat distant from the 0.2 µm threshold, the Enhance and PoGo systems can become a moderately acceptable option.

It should be noted that some studies have reported that the PoGo one step polishing system exhibited a similar or even better Ra when compared to the three step Sof-Lex Discs system [3,31,43]. According to the authors of these studies, the superior performance of PoGo could be attributed to the fine diamond powders used instead of aluminum oxide [3]. However, whenever the PoGo system was compared to the complete Sof-Lex Discs or four step systems, it was generally surpassed [38].

The manufacturer recommends the use of the Enhance system followed by the PoGo system for more favorable results [3]. However, this combination did not achieve good results, since they obtained an average Ra of 0.4631µm after being applied in 74 samples in five studies, with protocols between 20 and 30 s [32,33,34,36,37]. This situation does not necessarily mean a bad combination, since this result was obtained after calculating a general mean of all the results found and, in some of these investigations, the Ra was significantly higher for all participants [32,33,34]. In addition, in some Enhance PoGo studies, they obtained results below the Ra threshold of 0.2 µm and reached the heights of the Astropol and Sof-Lex Discs of three steps [36], and even managed to overcome the complete Sof-Lex Discs or four step systems, as happened in the study of Berger et al. [37]. Therefore, the manufacturer’s instructions must be followed and the Enhance/PoGo system should be used as one. Combining systems, as observed in this systematic review in most cases, did not seem to be very practical since it resulted in greater clinical complexity, requiring up to five steps per protocol [42], and did not always represent an improvement in the Ra, as can be seen in the study by Baseren et al. [46], where they combined the Astropol system, of proven efficiency, with Astrobrush and obtained an average Ra of 0.3450 µm, a value surpassed by the other systems evaluated in that research.

Regarding the composite resins reported in the different studies, it should be noted that although Filtek Supreme XT (transparent shade and dentin shade) and Tetric EvoCeram resin obtained the best results, the results obtained were averaged considering only 30 samples for each composite resin and these were obtained only from the study of Senawongse et al. [29], the results obtained after the composite resins with nanoparticles were subjected to the different polishing systems are highlighted in this study, with average values of Ra (µm) from 0.016 to 0.088 obtained. Therefore, it is evident that this study is one of the lowest reported surface roughness values, which suggests that Filtek Supreme XT and Tetric EvoCeram resins are not necessarily the best polished composite resins.

Within the limitations of this systematic review, there is great heterogeneity in the methodology of the selected studies, which makes it difficult to conduct a meta-analysis. However, it is considered a strength that all the included studies reported surface roughness in Ra units, which made it possible to average samples from different studies. For future systematic reviews related to the topic, it is recommended to evaluate the surface roughness of other materials, such as ceramics or metals, among others. Finally, it should be noted that the lacking of instruments for assessing the risk of bias or methodological quality specifically for in vitro studies is evident. For that, the research team decided to use standardized guidelines for improving quality and transparency in reporting in vitro studies in experimental dental research or dental materials [27,28]. New studies are needed, including variables such as the type and size of filling, degree of conversion, and the mechanical strength of several composite resins, to enable a complete discussion about the effectiveness of the polishing systems. Further systematic reviews in other fields, such as orthodontics, could elucidate the importance of polishing systems to ensure dental and periodontal health during and after the orthodontic treatment [15,16,17,18].

## 5. Conclusions

This systematic review reveals that the most effective polishing systems are those in which the size of the abrasive particle is systematically decreased, as is the case with the Astropol and Sof-Lex Discs system. Aluminum oxide is one of the most important components used to achieve a smooth surface. Likewise, diamond particles seem to be equally effective. Although the single step systems have evolved, they are still surpassed by multistep systems if simplicity and effectiveness are sought in the clinic. Similarly, according to the findings, it is advisable to opt for systems that have been demonstrated to be effective even with application times of a few seconds.

## Figures and Tables

**Table 1:** Application of guidelines for reporting preclinical in vitro studies on dental materials for the selected studies in the systematic review (n = 17).

First Author, Year	Item N° *													
	1	2	3	4	5	6	7	8	9	10	11	12	13	14
Senawongse, 2007 [29]	Yes	Yes	Yes	Yes	Yes	Yes	No	Yes	Yes	Yes	Yes	No	Yes	No
Ergucu, 2008 [30]	Yes	Yes	Yes	Yes	Yes	Yes	Yes	Yes	Yes	Yes	Yes	No	No	No
Ozel, 2008 [31]	Yes	Yes	No	Yes	Yes	Yes	Yes	Yes	Yes	Yes	Yes	Yes	No	No
Scheibe, 2009 [32]	Yes	Yes	Yes	Yes	Yes	Yes	Yes	Yes	Yes	Yes	Yes	No	No	No
Atabek, 2010 [33]	Yes	Yes	Yes	Yes	Yes	Yes	Yes	Yes	Yes	Yes	Yes	No	Yes	No
da Silva, 2010 [34]	Yes	Yes	Yes	Yes	Yes	No	Yes	Yes	No	Yes	Yes	No	Yes	No
Janus, 2010 [35]	Yes	Yes	No	Yes	Yes	Yes	Yes	Yes	Yes	Yes	Yes	Yes	No	No
Antonson, 2011 [36]	No	Yes	Yes	Yes	Yes	Yes	Yes	Yes	No	Yes	Yes	Yes	Yes	No
Berger, 2011 [37]	Yes	Yes	Yes	Yes	Yes	No	Yes	Yes	No	Yes	Yes	Yes	Yes	No
Buchgraber, 2011 [38]	Yes	Yes	Yes	Yes	Yes	No	Yes	Yes	No	Yes	Yes	No	Yes	No
Erdemir, 2012 [3]	Yes	No	Yes	Yes	Yes	Yes	Yes	Yes	No	Yes	Yes	Yes	No	No
Nunes, 2013 [39]	Yes	Yes	Yes	Yes	Yes	Yes	No	Yes	Yes	Yes	Yes	No	No	No
Avsar, 2015 [40]	Yes	Yes	Yes	Yes	Yes	Yes	No	Yes	Yes	Yes	Yes	No	No	No
Aytac, 2016 [41]	Yes	Yes	Yes	Yes	Yes	No	No	Yes	Yes	Yes	Yes	No	Yes	No
Yadav, 2016 [20]	No	Yes	Yes	Yes	Yes	Yes	Yes	Yes	Yes	Yes	Yes	No	No	No
de Carvalho Justo Fernandes, 2016 [42]	Yes	Yes	Yes	Yes	Yes	Yes	Yes	Yes	Yes	Yes	Yes	Yes	Yes	No
Alfawaz, 2017 [43]	Yes	Yes	No	Yes	Yes	Yes	Yes	Yes	Yes	Yes	Yes	No	No	No

* Item 1: summary; Item 2: Introduction, background and objectives; Item 3: methods, intervention; Item 4: results; Item 5: sample size; Item 6: randomization, generation of sequences; Item 7: allocation concealment mechanism; Item 8: implementation; Item 9: blinding; Item 10: statistical methods; Item 11: results and estimation; Item 12: discussion, limitations; Item 13: other information, financing; Item 14: protocol.

**Table 2:** Main characteristics of the studies included in the systematic review (n = 17).

First Author, Year	Country	Type of Resin (TR)	Resin Manufacturer (RM)	Finishing System (FS)	Polishing System (PS)	PS Manufacturer
Senawongse, 2007 [29]	Thailand	Tetric Ceram	Ivoclar Vivadent	N.I	Sof-Lex Discs	3M ESPE
		Filtek Z350	3M ESPE		Astropol	Ivoclar Vivadent
		Filtek Supreme XT (dentin shade)	3M ESPE			
		Filtek Supreme XT (transparent shade)	3M ESPE			
		Estelite Sigma	Tokuyama, Tokyo, Japan			
		Tetric EvoCeram	Ivoclar Vivadent			
		Ceram X	Dentsply			
		Premise	Kerr Corporation			
Ergucu, 2008 [30]	Turkey	Filtek Supreme	3M ESPE	320 grit silicon carbide paper	PoGo	Dentsply
		Grandio	Voco		OptraPol	Ivoclar Vivadent
		Tetric Ceram	Ivoclar Vivadent		One Gloss	Shofu Dental Corp
		Premise	Kerr Corporation			
		Ceram X	Dentsply			
Ozel, 2008 [31]	Turkey	Filtek Supreme	3M ESPE	1200-grit silicon carbide paper.	PoGo	Dentsply
		Ceram X	Dentsply		OptraPol	Ivoclar Vivadent
		Aelite Aesthetic Enamel	Bisco, Inc. Schaumburg, IL, USA		Sof-Lex Discs	3M Espe
		Tetric Ceram	Ivoclar Vivadent			
		Grandio	Voco			
Scheibe, 2009 [32]	Brazil	Charisma Diamond	Heraeus Kulzer	Fine grit diamond burs and extra-fine diamond burs	Sof-Lex Discs	3M Espe
		Fill Magic^®^	Vigodent, Rio de Janeiro, RJ, Brazil		Enhance + PoGo	Dentsply
		TPH Spectrum^®^	Dentsply		Felt disks + diamond paste	FGM Ind.
Atabek, 2010 [33]	Turkey	Ceram X	Dentsply	Carbide burs	Enhance + PoGo	Dentsply
		Clearfil Majesty Esthetic	Kuraray		Lasting Touch	
					Enhance + PoGo + nanotechnology liquid polish (lasting touch)	
da Silva, 2010 [34]	Brazil	Filtek Z350	3M ESPE	Extrafine diamond tip No 3195F	Enhance + PoGo	Dentsply
Janus, 2010 [35]	France	Filtek Supreme	3M ESPE	N.I	Sof-Lex Discs	3M Espe
		Grandio	Voco		CompoSystem	Komet, Gebr. Brasseler GmbH&Co (Lemgo, Germany)
		Synegy D6	Coltene Whaledent AG (Altstatten, Switzerland)			
		Tetric Ceram	Ivoclar Vivadent			
Antonson, 2011 [36]	USA	Filtek Supreme	3M ESPE	1200 grit size sand paper	Astropol	Ivoclar Vivadent
					Enhance + PoGo	Dentsply
					Sof-Lex Discs	3M Espe
					EXL-695	3M Espe
Berger, 2011 [37]	Brazil	Filtek Supreme	3M ESPE	N.I	Sof-Lex Discs	3M Espe
					FlexiDisc and Enamelize	Cosmedent Inc
					Enhance + PoGo	Dentsply
Buchgraber, 2011 [38]	Austria	Filtek Supreme	3M ESPE	Low speed fissure bur (green, 3000 rev/min) for 10 s	PoGo	Dentsply
					Sof-Lex Discs	3M Espe
Erdemir, 2012 [3]	Turkey	Filtek Supreme	3M ESPE	1200 grit silicon carbide paper.	PoGo	Dentsply
		Ceram X	Dentsply		Sof-Lex Discs	3M Espe
		Grandio	Voco			
Nunes, 2013 [39]	Brazil	Charisma Diamond	Heraeus Kulzer	N.I	Sof-Lex Discs	3M ESPE
		Premise	Kerr Corporation		Enhance	Dentsply
		Filtek Supreme	3M ESPE		Fine grit diamond burs 30μm and extra-fine diamond burs 20 μm + Felt disks + diamond paste	KG Sorensen / FGM Ind.
Avsar, 2015 [40]	Turkey	Grandio	Voco	1000 grit silicon carbide paper	Sof-Lex Discs	3M Espe
		Ice	SDI, Victoria, Australia		Silicone rubber Kerr	Kerr
		Smile	Pentron, Wallingford, CT, USA		12 then 30 fluted carbide finishing bur	KG, Sorensen, Barueri, Brazil
		Aelite Enamel	Bisco, Inc. Schaumburg, IL, USA		Fine then extra-fine diamond finishing bur	KG, Sorensen, Barueri, Brazil
		Premise	Kerr Corporation			
		Filtek Supreme	3M Espe			
Aytac, 2016 [41]	Turkey	Estelite Ʃ Quick	Tokuyama, Tokyo, Japan	1000 grit silicon carbide paper.	Occlubrush	Kerr
		Clearfil Majesty Esthetic	Kuraray		Clearfil Twist Dia	Kuraray
		Zenit	President		Sof-Lex Spiral Finishing & Polishing Wheels	3M Espe
		Filtek Z550	3M Espe		Sof-Lex Discs	3M Espe
Yadav, 2016 [20]	India	Ceram X	Dentsply	diamond burs 50 µm + Super Snap Rainbow Technique Kit VIOLET	Super-snap Rainbow Technique Kit	Shofu Dental Corp
				diamond burs 50 µm	Sof-Lex Discs	3M Espe
				diamond burs 50 µm + Enhance, tip shape.	Enhance + discs impregnated with Gloss Prisma paste.	Dentsply
de Carvalho Justo Fernandes, 2016 [42]	Brazil	Evolu-X	Dentsply	N.I	Sof-Lex Discs	3M ESPE
					Sof-LexTM Pop-On + felt disc associated to diamond paste (Diamond GlossTM)	KG, Sorensen, Barueri, Brazil
					Sof-LexTM Pop-On + Astrobrush	Ivoclar Vivadent
					Práxis TDV	TDV Dental Ltd.a.
					Práxis TDV + felt disc associated to diamond paste (Diamond GlossTM)	KG, Sorensen, Barueri, Brazil
					Práxis TDV + Astrobrush	Ivoclar Vivadent
Alfawaz, 2017 [43]	Saudi Arabia	Filtek Z350	3M ESPE	600 grit silicon carbide paper	PoGo	Dentsply
		Ceram X	Dentsply		Sof-Lex Discs	3M Espe

N.I: No information available.

**Table 3:** Summary of the polishing protocols (PP) according to the studies provided in the systematic review (n = 17).

Polishing System (PS)	Polishing Protocol (PP)	Number of Steps Polishing System (SPS)	Total Samples (TS)	Average in Microns According to the Protocol (RA)
**12 then 30 fluted carbide finishing bur**	N.I	2	48	0.3713
**Astropol**	20 s/---rpm	3	5	0.1200
	60 s/12,000 rpm		80	0.0500
**Clearfil Twist Dia**	45 s/10,000 rpm	2	40	0.2000
**CompoSystem**	20 s/Medium 10,000 rpm, Fine 1000 rpm, Ultrafine 10,000 rpm	3	24	0.1520
**Enhance**	20 s/---RPM	1	30	0.2167
**Enhance + CUP shape Prisma Gloss paste**	30 s/---rpm	3	10	0.1457
**Enhance + PoGo**	30 s/20,000 rpm	2	10	0.8400
	20 s/---rpm		17	0.3240
	N.I		20	0.7250
	30 s/---rpm		27	0.4960
**Enhance + PoGo + nanotechnology liquid polish (lasting touch)**	N. I	3	20	0.675
**EXL-695**	20 s/---rpm	2	5	0.1200
**Felt disks + diamond paste**	60 s/---rpm	1	27	0.6396
**Fine grit diamond burs 30 μm and extra-fine diamond burs 20 μm + Felt disks + diamond paste**	20 s/---RPM	3	30	0.2933
**Fine then extrafine diamond finishing bur**	N.I	2	18	10.350
**FlexiDisc and Enamelize**	30 s/20,000 rpm	4	10	0.1260
**Lasting Touch**	N.I	1	20	0.9850
**Occlubrush**	45 s/10,000 rpm	1	20	0.5933
**One Gloss**	30 s/15,000 rpm	1	25	0.4606
**OptraPol**	30 s/15,000 rpm	1	75	0.3289
**PoGo**	30 s/---rpm	1	21	0.2360
	10 s/---rpm		5	0.5600
	30 s/15,000 rpm		95	0.201
**Práxis TDV**	30 s/---rpm	4	5	0.2080
**Práxis TDV + Astrobrush**	30 s/---rpm	5	5	0.2100
**Práxis TDV + felt disc associated to diamond paste (Diamond GlossTM)**	30 s/---rpm	5	5	0.1320
**Silicone rubber Kerr**	N. I	2	48	0.3907
**Sof-Lex Discs**	10 s/---rpm	4	5	0.1450
	30 s/20,000 rpm		10	0.1000
	20 s/Coarse 10,000 rpm, Medium 10,000 rpm, Fine 30,000 rpm, Superfine 30,000 rpm		24	0.1213
	60 s/12,000 rpm		80	0.0479
	20 s/---rpm		30	0.1800
	30 s/---rpm		5	0.1860
	N.I	3	10	0.0430
	45 s/10,000 rpm		40	0.1725
	30 s/20,000 rpm with water cooling		48	0.3843
	30 s/---rpm		21	0.2433
	30 s/15,000 rpm		70	0.2188
	20 s/---rpm		32	10.266
**Sof-Lex Spiral Finishing & Polishing Wheels**	45 s/10,000 rpm	3	40	0.2475
**Sof-LexTM Pop-On + Astrobrush**	30 s/---rpm	5	5	0.1400
**Sof-LexTM Pop-On + felt disc associated to diamond paste (Diamond GlossTM)**	30 s/---rpm	5	5	0.1120
**Super-snap Rainbow Technique Kit**	N.I	3	10	0.0799

## Data Availability

Not applicable.
